# Genetic analysis of agronomic and physiological traits associated with latex yield revealed complex genetic bases in *Hevea brasiliensis*

**DOI:** 10.1016/j.heliyon.2024.e33421

**Published:** 2024-06-22

**Authors:** Sigit Ismawanto, Martini Aji, David Lopez, Pierre Mournet, Eric Gohet, Afdholiatus Syafaah, Florelle Bonal, Fetrina Oktavia, Taryono, Siti Subandiyah, Pascal Montoro

**Affiliations:** a CIRAD, UMR AGAP Institut, F-34398, Montpellier, France; b UMR AGAP Institut, Univ Montpellier, CIRAD, INRAE, Institut Agro, F-34398, Montpellier, France; c Faculty of Agriculture, Universitas Gadjah Mada, Bulaksumur, Sleman, Yogyakarta, 55281, Indonesia; d Pusat Penelitian Karet, Sembawa, Banyuasin, Sumatera Selatan, 30953, Indonesia; e CIRAD, UMR ABsys, F-34398, Montpellier, France

**Keywords:** Clonal typology, Inorganic phosphorus, Latex diagnosis, Latex yield, QTL, Rubber, Sucrose

## Abstract

*Hevea brasiliensis,* a natural rubber producing species, is widely cultivated due to its high rubber yield potential. Natural rubber is synthesised in the rubber particles of laticifers. Latex diagnosis (LD) was established to characterise the physiological state of the laticiferous system by measuring its physiological parameters, i.e., sucrose, inorganic phosphorous (Pi), thiols and total solid content (TSC). Rubber clones are often classified in three groups i.e., quick starters, medium starters and slow starters. To better understand the genetic bases of latex yield, a biparental population was generated from a cross between the quick-starter clone PB 260 and the medium-starter clone SP 217. LD was performed during the peak latex production season and used to calculate sucrose loading. The agronomic and physiological parameters associated with latex yield led to the classification of genotypes according to the rubber clonal typology and to the identification of quantitative trait loci (QTL) using a high-density map. Inorganic phosphorous content was positively associated with yield during the first year of production thus enabling identification of quick-starter clones. In addition, the LD-based clonal typology led to determine the long-term yield potential and the use of appropriate ethephon stimulation. QTL analysis successfully identified several QTLs related to yield, sucrose, Pi and TSC. One QTL related to sucrose loading was identified in the same position as the QTL for sucrose on linkage group 1. To our knowledge, this is the first study to report QTL analysis for this trait. The use of a high-density map enables the identification of genes underlying QTLs. Several putative genes underlying QTLs related to yield, sucrose and TSC were identified.

## Highlights

• Sucrose, inorganic phosphorus and yield allow identifying quick-starter clones.
• QTL analysis revealed a complex genetic basis of latex production.
• First application of latex diagnosis-based clonal typology in breeding.
• First identification of a QTL associated with sucrose loading.
• First identification of chromosomal regions underlying QTLs linked to physiological parameters.

## 1 Introduction

*Hevea brasiliensis* is the most widely cultivated natural rubber-producing species thanks to its high rubber yield potential, its greater adaptability to different climatic conditions, the quality of its rubber and its molecular characteristics, which are not easily imitated by synthetic rubber [1,2]. Asian countries produce about 90 % of the world's natural rubber, and Indonesia is the second largest natural rubber producer after Thailand [3].

The prolonged low price of rubber has led smallholders to reduce their tapping activities and farm maintenance, and even to abandon rubber farming altogether. This situation has reduced both the rubber plantation area and rubber production in Malaysia [4], Indonesia [5,6], and China [7]. In some cases, high production costs and low rubber prices have led to overexploitation of rubber trees, especially in estate plantations [8].

Latex is produced and stored in specific cells in the bark called laticifers and harvested by tapping [9]. Natural rubber is synthesised in the rubber particles of laticifers. It was estimated that the maximum yield potential of rubber trees could theoretically reach 9600 kg of rubber/ha/year [10]. However, the yield of the rubber clones recommended in Indonesia ranges from 1500 to 2500 kg/ha/year [11]. There is therefore still a significant gap between the maximum rubber yield potential and the yields observed in plantations.

One important factor that affects latex production is tapping panel dryness (TPD), a physiological disorder that hampers the flow of latex after tapping. This is due to the agglutination of rubber particles associated with the bursting of lutoids induced by oxidative stress [12]. Monitoring the dry cut length (DCL) appeared to be a simple method to identify TPD-susceptible clones [13]. Early TPD occurrence was induced by high intensity harvesting [14].

Latex diagnosis (LD) was used to observe the physiological state of the laticifer system by measuring the following latex physiological parameters, sucrose content (Suc), inorganic phosphorous content (Pi), thiol content (RSH), magnesium (Mg), total solid content (TSC), pH, redox potential, and the bursting index [15]. The ability of latex cells to produce rubber depends on their sucrose and inorganic phosphorus contents. Sucrose content reflects the balance between the transfer of sucrose from the apoplast to the latex cells and the consumption of sucrose by latex cells for energy production and metabolic activity, including rubber biosynthesis. Pi indicates the intensity of metabolic activity in latex cells [16,17]. RSH reflects the protective capacity of lutoid membranes and prevention of TPD [18,19]. Four main parameters (Suc, Pi, TSC, RSH) of LD have been used for decades in estate plantations to obtain the optimum latex yield and to prevent TPD.

Generally, rubber clones are classified in three main groups i.e., quick starters, medium starters and slow starters. Quick-starter clones show a high metabolic activity (high Pi) and high latex production during the first years of tapping, resulting in the overconsumption of sucrose. These clones usually have a low long-term latex yield potential and a limited response to ethephon stimulation [17]. By contrast, slow-starter clones have low metabolic activity (low Pi) and low initial production leading to the accumulation of sucrose (high Suc). These clones can be activated by ethephon stimulation to increase the yield, and their long-term yield potential is consequently higher.

An attempt has been made to classify rubber clones in order to predict the yield potential and the most appropriate harvesting system. The first rubber clonal typology was established with many physiological parameters including sucrose, RSH, Pi, Mg, pH, bursting index, and redox potential and led to three groups of rubber clones [18]. In 1995, the clonal typology was simplified by reducing it to only four main parameters, sucrose, dry rubber content (DRC), Pi and RSH [20]. A correlation was shown between yield and metabolic activity and sucrose. Sucrose content is the result of sucrose loading into laticifers and its consumption for rubber biosynthesis, in which all these parameters depend on metabolic activation. The current classification of rubber clones is performed in a two-dimensional matrix containing five different metabolic classes and three different sucrose loading classes [21]. Of the fifteen possible classes identified to date, all recommended rubber clones are classified in thirteen classes.

The detection of a quantitative trait locus (QTL) is based on the identification of the associations between certain genetic markers and traits (phenotype), thereby revealing the existence and location of important genes on chromosomes. Genetic markers associated with QTLs can be used to shorten the selection process by implementing marker-assisted selection [[22], [23], [24]]. QTL analyses using various types of genetic markers (RFLP, AFLP, SSR, SNP) have been done in *Hevea* for some traits including growth, disease resistance and latex yield [[25], [26], [27], [28], [29], [30], [31], [32], [33]]. More recently, candidate genes underlying QTLs were identified for growth [32] and dry latex yield [33].

The aim of the present study was to identify the genetic bases of physiological parameters associated with latex production. A biparental population was generated from a cross between two rubber clones, PB 260, a quick-starter clone, and SP 217, a medium-starter clone. A high-density map of 3106 single nucleotide polymorphism markers (SNPs) was established by genotyping-by-sequencing. Latex yield was monitored for one year, while LD (Suc, Pi and TSC) was conducted in the peak latex production season. These agronomic and physiological parameters associated with latex yield enabled the classification of genotypes and the identification of QTLs for each LD parameter. Analysis of the chromosomal regions underlying the QTLs revealed several candidate genes potentially associated with functions linked to latex production.

## 2 Materials and Methods

### 2.1 Establishment of the biparental population

The present study started in 2012 with the establishment of a biparental population generated by hand-pollination between rubber clones PB 260 and SP 217. Each seedling represented one genotype. Each genotype was grafted onto a clone GT1 illegitimate seedling as rootstock and used as budwood in a dedicated budwood garden for the propagation of planting material by grafting at the Sembawa Experimental Garden, Indonesian Rubber Research Institute, Sembawa, Banyuasin District, South Sumatera Province, Indonesia (GPS coordinates: 2°55′58.6″S 104°32′07.0″E). A paternity test was carried out on all seedlings at CIRAD in France to confirm their legitimacy using a combination of 8 SSR markers.

### 2.2 Establishment of a small-scale clone trial

Two-year-old seedlings were used as source of budwood (scion) to produce a minimum of 10 grafted plants per genotype growing in polybag. Two hundred and one selected genotypes were planted in November 2016 at the Sembawa Research Centre (GPS coordinates: 2°58′13.2″S 104°28′51.1″E) in a small-scale clone trial (hereafter termed SSCT1). The experimental design consisted of blocks with five replicates and two trees per genotype per replicate. Two parent clones (PB 260 and SP 217) and six recommended clones (GT1, AVROS 2037, RRIC 100, PR 261, IRR 39 and IRR 112) were used as control. The conformity of the planting material used in SSCT1 was checked at CIRAD (see below). Twelve genotypes were discarded from the analysis because the result of their conformity test was negative.

### 2.3 Conformity test and construction of a high-density map

#### 2.3.1 DNA extraction

Genomic DNA was extracted using an automated method with a mixed alkyl trimethylammonium bromide buffer (MATAB) and NucleoMag Plant Kit (Macherey-Nagel, Düren, Germany) as described in Cormier et al. [34]. Genomic DNA was quantified with a Fluoroskan Ascent FL fluorometer (Thermo Fisher Scientific, Waltham, MA, USA) and quality was checked using agarose gel electrophoresis.

#### 2.3.2 Genotyping with SSR markers of the biparental population and SSCT1 planting material

The individuals from the biparental family of PB260 x SP217 were genotyped with 279 SSR, including SSR-enriched genomic libraries and SSR-containing EST or SSH cDNA sequences, while the clonal identities of the planting material used in SSCT1 were checked using a set of 8 SSR [[35], [36], [37]]. Migration of PCR products was performed on an ABI 3500xL Genetic Analyser (Life Technologies, Carlsbad, CA, USA). Alleles were scored using GeneMapper v.4.1 software (Applied Biosystems).

#### 2.3.3 Preparation for genotyping-by-sequencing

A genomic library was prepared using *Pst*I-*Mse*I (New England Biolabs, Hitchin, UK) restriction enzymes with a normalized 200 ng quantity of DNA per sample [38]. Next generation sequencing (NGS) was conducted on an Illumina Novaseq system (150-bp, paired-end reads) at the MGX platform in Montpellier, France.

#### 2.3.4 SNP calling and filtering

SNP calling, filtering and matrix correction were carried out using the same procedure as described by Mournet et al. [39]. Briefly, genotyping-by-sequencing (GBS) sequences were demultiplexed and their adapters trimmed using GBSX v1.2 and cutadapt v3.5. SNPs were called from the cleaned sequences using the vcfhunter pipeline. Reads were done on one haplotype of the clone PB 260 obtained using the long-read sequencing PacBio methodology (see § 2.8). Variants were filtered using several criteria: sites with read depths of <10 and >3000 were removed, variant sites with >20 % missing genotype calls and minor allele frequencies per site <10 % were filtered out; minor allele frequencies per site >10 % and only biallelic sites were kept.

#### 2.3.5 Linkage analysis and construction of the parental map

The genetic map was made using SSR and SNP markers with different modules of LepMAP3 software version 0.4 [40]. The optimal number of 18 linkage groups (LG) was obtained using a LOD threshold of 19 and a marker size limit per group of 40 from the SeparateChromosomes2 module. JoinSingles2 was run to assign additional markers to existing linkage groups using decreasing LOD score iterations from 18 to 5. The genetic distances were calculated using the OrderMarkers2 module with the Kosambi function. Following the recommendations made by the software developer, all markers that created large gaps at the top or bottom part of the LGs were discarded. Concerning the numbering and orientation of the LGs, the same nomenclature as that used by Lespinasse and collaborators was applied thanks to the use of the same SSR markers in the two studies [26].

### 2.4 Yield determination

The trees in the SSCT1 trial were opened on January 4, 2021, using half-spiral tapping and a tapping frequency of once every three days. This tapping system is noted S/2 d3 7d/7 according to the Vijayakumar and collaborators standard [41]. Data on yield in the form of a cup lump (latex that coagulated naturally in the collecting cup) were collected one day after tapping. The fresh weight of the cup lump was recorded. The dry rubber content of the cup lump was measured to calculate the dry rubber yield.

$$\text{Dry}\;\text{rubber}\;\text{content}\;\left(\text{DRC}\right)=\frac{\text{Dry}\;\text{weight}\;\text{of}\;\text{cup}\;\text{lump}}{\text{Fresh}\;\text{weightof}\;\text{cup}\;\text{lump}}x100\%$$

Yieldpertreepertapping=CuplumpfreshweightxDRC

Yield data in grammes of rubber per cm tapping cut were used to minimise the effect of different tree trunk sizes.

$$\text{Yield}\;\text{per}\;\text{cm}\;\text{of}\;\text{the}\;\text{tapping}\;\text{cut}\;\text{per}\;\text{tapping}=\frac{\text{Yield}\;\text{per}\;\text{tree}\;\text{per}\;\text{tapping}}{\text{Length}\;\text{of}\;\text{the}\;\text{tapping}\;\text{cut}\;\left(\text{cm}\right)}$$

### 2.5 Latex diagnosis

The latex diagnosis was carried out in April 2021 during the peak rubber production period in South Sumatera, Indonesia. To be able to analyse all the latex diagnosis samples from one replicate in one day, the latex from the two trees per genotype per replicate were pooled to obtain 4 ml of fresh latex for each sample. Consequently, one sample per genotype was analysed for each replicate. Half a millilitre of fresh latex was mixed with 4.5 ml of 2.5 % TCA +0.1 % EDTA buffer and then filtered through filter paper to obtain clear serum solution. The serum solution was then used for analysis. Sucrose content was analysed using the anthrone method [42]. One hundred microlitres of latex serum were placed in a 20-ml test tube containing 3 ml of anthrone reagent and incubated in a boiling water bath for 15 min. After cooling, the solution was mixed thoroughly and the absorbance of the solution was measured at a wavelength of 620 nm. Pi content was analysed with ammonium molybdate [43]. Half a millilitre of latex serum was placed in a 20-ml test tube containing 1.5 ml of 2.5 % TCA buffer. Two millilitres of ferrous sulphate-ammonium molybdate reagent was added and the solution was mixed thoroughly. The absorbance of the solution was measured at a wavelength of 740 nm with a spectrophotometer (SPECTROstar Nano, BMG LABTECH, Ortenberg, Germany). The TSC of the latex sample was measured by weighing 2 g of fresh latex in an aluminium disc and then oven dried at 90 °C until constant weight and then weighed again. TSC was obtained by calculating the percentage of dried rubber weight and the fresh weight.

$$\text{Total}\;\text{Solid}\;\text{Content}\;\left(\text{TSC}\right)=\frac{\;\text{Latex}\;\text{dried}\;\text{weight}}{\text{Latex}\;\text{fresh}\;\text{weight}}x\;100\%$$

### 2.6 Clonal typology

Clonal typology was analysed on the basis of sucrose and Pi contents obtained from the average LD and the average latex yield per centimetre of tapping cut per tapping during a tapping year. Sucrose loading was calculated based on the linear regression residuals between sucrose-yield and sucrose-Pi [21]. The average value of the residuals from the two linear regressions was used to form three classes of metabolic activity (low, medium, and high). The five metabolic classes were obtained using quantile classification of Pi values (low = 0–10 %, low-medium = 10–25 %, medium = 25–75 %, medium-high = 75–90 % and high = 90–100 %). The three sucrose loading classes and the five metabolic classes were then used to group the genotypes in a two-dimensional matrix table.

### 2.7 Data analysis, heritability calculation and QTL detection

The data of all the observed variables were analysed with Microsoft Excel (Washington, USA) and XLStat software (Denver, USA). The PCA was performed with LS-means calculated from the value of the five replicates, each replicate being the average of the values of two trees per genotype. The heritability of all the observed variables was calculated with the lme4 package in R [44]. The blupline value from the heritability calculation was used for QTL analysis with MapQTL version 6 (MapQTL6, Wageningen, Netherlands). Since the data did not follow a normal distribution, the non-parametric Kruskal–Wallis (KW) test was applied [45].

### 2.8 Identification of genes underlying QTLs

A new genome assembly for clone PB 260 was used to identify the putative genes involved in the traits under study. The 1.6 Gb of assembled contigs obtained from HiFi PacBio reads is publicly available at https://zenodo.org/doi/10.5281/zenodo.10281548. Putative coding genes were determined using the full length cDNA sequences of Reyan7-33-97 annotation [46]. The robustness of these predictions was tested on several gene families [47,48]. Sequences were aligned using minimap2 v.2.22 and the “splice” preset [49]. Transcripts were then obtained from spliced alignments using UCSC Kent tools v385 bedToGenePred followed by genePredToGtf. The QTL regions on PB 260 contigs and gene prediction gtf files were intersected using Bedtools v2.30.0 [50].

## 3 Results

### 3.1 The biparental population derived from clones PB 260 and SP 217 exhibited highly variable yield, sucrose, Pi, and TSC

**Fig. 1:**
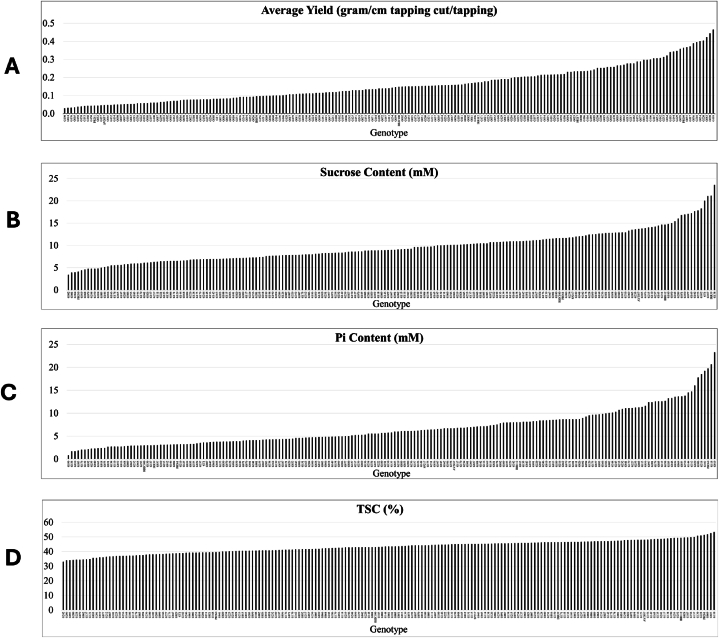
Distribution of average yield in grammes of dry rubber per centimetre of tapping cut per tapping (A), Suc (mM) (B), Pi (mM) (C) and TSC (%) of 201 genotypes derived from the cross between clones PB 260 and SP 217 and eight control clones.

The variables observed during the first year of tapping showed that the average yield per centimetre tapping cut per tapping in one year of tapping ranged from 0.03 to 0.46 g of rubber per cm of tapping cut per tapping (Fig. 1A, Supplementary data 1). Among the control clones, the parent clones, PB 260 and SP 217, were classified as high and medium-high yielding clones, respectively, for the first year of tapping, while PR 261 was the lowest yielding clone. Sucrose content ranged from 3.4 mM to 23.6 mM per ml of latex. (Fig. 1B). Among the control clones, IRR 39 had the highest sucrose content (21.1 mM) followed by GT1 (20.0 mM) while the parent clone, SP 217, had medium-high sucrose content (11.8 mM) and PB 260 had the lowest sucrose content (4.1 mM).

Pi content ranged from 0.8 mM to 23.3 mM (Fig. 1C). Among the controls, the parent clone, PB 260, had the highest Pi content (19.7 mM), and SP 217 had medium Pi content (6.3 mM). The control clone with the lowest Pi content was RRIC100 (3.0 mM). TSC ranged from 33 % to 53.3 % (Fig. 1D). Parent clones PB 260 and SP 217 had 39.6 % and 45.2 % TSC respectively.

### 3.2 Principal component analysis of variables observed during the first year of tapping

**Fig. 2:**
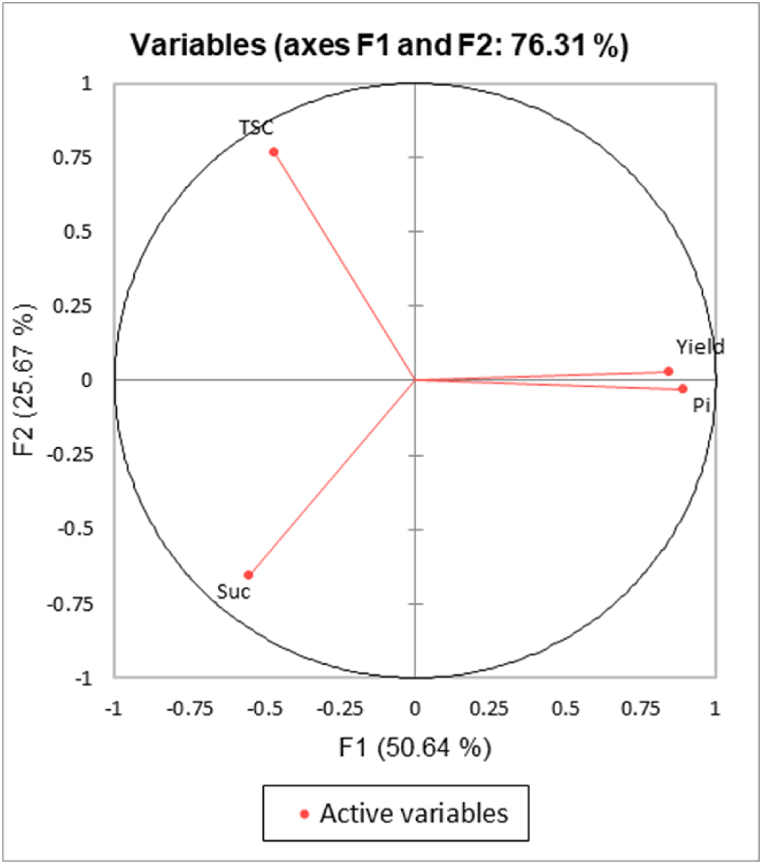
Principal component analysis of yield (gm rubber/cm/tapping), Suc (mM), Pi (mM), and TSC (%).

PCA was performed using LS means of variables observed for each genotype (Fig. 2). The F1 axis explains 50 % of total variability. Pi and yield are located opposite Suc and TSC. Only Pi and yield showed a strong positive correlation. The correlations between variables and factors were 0.89 and 0.84, respectively. However, the linear regression showed no significant correlation (R2 = 0.438; Supplementary data 2). On the F2 axis, Suc is located opposite TSC, and the correlations between variables and factors were −0.66 and 0.77, respectively. This negative correlation was not confirmed by linear regression analysis either.

### 3.3 Classification of genotypes

The genotypes and control clones were classified based on the latex yield using quantile estimation (Table 1 and Supplementary data 3). The parent clones, PB 260 and SP 217, belong to high yield classes 6 and 5, respectively, while the other control clones were classified as belonging to class 1 to class 4. Classes 3 and 4 accounted for half the population (96 genotypes).

**Table 1:** Classification of control clones and genotypes based on the average of one-year latex yield data (gm rubber/cm/tapping). Yield classes range from 1 to 6 with genotypes in class 1 produce the lowest yield and those in class 6 the highest.

Yield class	Control clone	Number of genotypes
1	AVROS 2037 & PR 261	17
2	GT1	29
3	IRR 39	48
4	IRR 112 & RRIC 100	48
5	SP 217	28
6	PB 260	19

Clones that produce high yields the first year do not necessarily continue to produce high yields in the long run. They are high metabolism clones that do not require ethephon stimulation, while some other clones with low metabolic activity require ethephon stimulation. Clonal typology analysis was carried out to predict the potential increase in yield under stimulation based on the initial yield, sucrose loading, and metabolic activity related to Pi. The classification was undertaken in three sucrose loading classes and five metabolic activity classes (Table 2, Supplementary data 4).

**Table 2:** Number of genotypes from the progeny and position of control clones for each typology group calculated based on their latex diagnosis.

		Metabolism				
		Low	Low-Medium	Medium	Medium-High	High
Sucrose Loading	Low	5	7	34	14	6, PB 260
	Medium	7	8,	33	11	5
			PR261			
	High	7	11,	29,	5	7
			GT1, RRIC 100, IRR 39	AVROS 2037, IRR 112, SP 217		

The two-dimensional matrix table shows genotypes for each typology group. The female parent clone PB 260 has a high metabolism and low sucrose loading while the male parent SP 217 has a medium metabolism and high sucrose loading. The SP 217 clone can be stimulated to activate metabolism and consume available sucrose. Ninety-six of the genotypes have a medium metabolism but with different levels of sucrose loading, revealing that genotypes with medium and above all high sucrose loading can be activated by ethephon stimulation. The genotypes with high sucrose loading and a low to medium metabolism (47 genotypes) have the capacity to increase their yield if suitable ethephon stimulation is used (Supplementary data 4). By contrast, genotypes with medium-high and high metabolism have reached their full production potential and even those with high sucrose loading cannot be stimulated to improve their yield.

### 3.4 Heritability analysis

Heritability was calculated for all the variables i.e., yield, sucrose, Pi, and TSC (Table 3). All the traits showed high heritability, i.e. ranging from 0.92 for yield and up to 0.79 for sucrose.

**Table 3:** Heritability of yield (gm/cm/tapping), sucrose (mM), Pi (mM) and TSC (%). GV = genetic variance, Ve = residual variance, hi = heritability at the level of the individual tree (GV/(GV + Ve)), hf = heritability at the level of clonal estimations (GV/(GV + Ve/n), where n is the number of trees per genotype).

Variables	GV	Ve	hi	hf
Yield	72.83	31.79	0.69	0.92
Sucrose	8.32	10.62	0.44	0.79
Pi	14.06	13.31	0.51	0.84
TSC	15.19	16.31	0.48	0.81

### 3.5 Genetic mapping based on genotyping-by-sequencing

A total of 105,993 SNP markers were identified in the parental individuals and mapping population. The filters described in Materials and Methods reduced the number of SNPs to 7374. Some markers were removed as they were redundant. With 3106 SNPs, 273 SSR markers and 183 individuals, the map spanned a total length of 2046.294 cM with an average distance between markers of 0.60 cM (Supplementary data 5). Compared to previous studies, this map is the smallest ever published: Lespinasse et al. [26] (2144 cM), Souza et al. [25] (2688.8 cM) and Munyengwa et al. [38] (2600.9 cM & 2215.1 cM), despite the significant number of markers identified. Linkage analysis revealed 18 linkage groups corresponding to the number of haploid chromosomes in the rubber tree. The largest linkage group (LG) was 10 (144.913 cM), the smallest was 17 (88.259 cM), and the number of markers per linkage group ranged from 140 (LG2) to 266 (LG10). The highest marker density was found in LG15 (the distance between markers was 0.533 cM, and the maximum distance between markers was 2.522 cM). In contrast, the lowest density was found in LG2 (the distance between markers was 0.813 cM, and the maximum distance between markers was 10.277 cM).

**Fig. 3:**
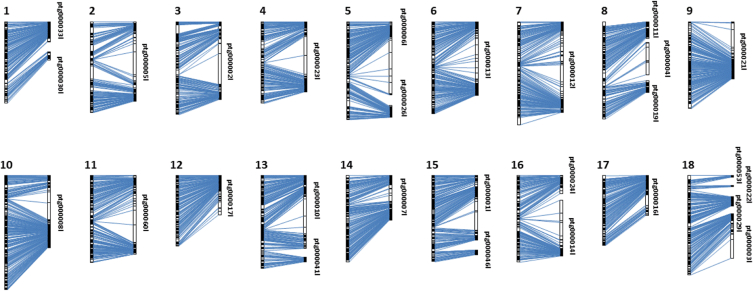
Analysis of collinearity between the genetic map and the physical map of the 18 *Hevea brasiliensis* linkage groups in the PB260 x SP217 cross. The vertical scale on the left represents the linkage group and on the right, the PB 260 assembled contigs obtained from HiFi PacBio reads (https://zenodo.org/doi/10.5281/zenodo.10281548); 1 cM = 1 Mb.

To assess the quality and accuracy of the genetic map, the locations of the markers on the genetic map were compared with the genome assembly for clone PB 260 (Fig. 3). Perfect collinearity was observed between the genetic map and the corresponding chromosome or scaffold. Centromeric regions are easily observed and certain contigs show complete assembly at the scale of a chromosome or of the arm of a chromosome.

### 3.6 QTL detection

**Fig. 4:**
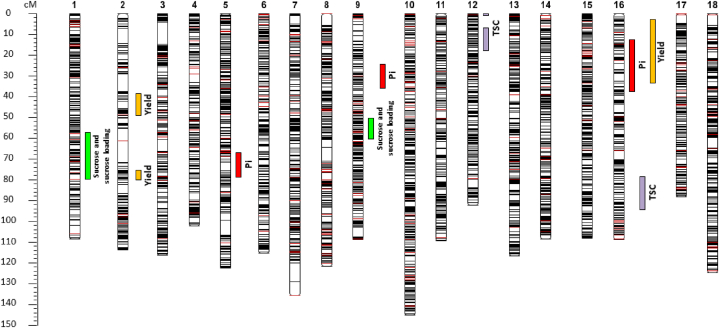
QTLs for yield, sucrose, sucrose loading, Pi, and TSC using a high-density genetic map based on a Kruskal-Wallis (K*) test. The red and black bars correspond to SSR and SNP markers, respectively.

A normality test was conducted on the data before heritability was calculated. Not all the variables observed followed a normal distribution, so QTL analysis was performed using the Kruskal-Wallis’ test (Supplementary data 6, 7, 8, 9 and 10). Table 4 summarises the position of markers with high K* and low p-values. Two QTLs related to sucrose content were identified on LG 1 and LG 9 (Fig. 4). One QTL related to sucrose loading was identified on LG 1, in the same position as the QTL for sucrose. To our knowledge, this is the first study to conduct QTL analysis for this trait. Three QTLs related to inorganic phosphorous content were located on LG 5, LG 9 and LG 16, respectively. QTLs related to yield were identified on LG 2 and LG 16. Two QTLs on LG 12, and one QTL on LG 16 were correlated with TSC. Interestingly, QTLs for yield and Pi on LG 16 are closely contiguous (position 7.443 cM and 22.035 cM), and also associated with TSC on the same LG (position 90.602 cM).

**Table 4:** Summary of significant QTLs identified for yield, sucrose, sucrose loading, Pi and TSC on different observation dates calculated with Kruskal-Wallis test. LG: linkage group, SNP: single nucleotide polymorphism, K*: the Kruskal-Wallis’ test statistic K*.

Trait	Observation	LG	Marker	Position (cM)	K*	P-value.
Yield	2021	2	g2T2094	42.404	20.626	0.0001
		2	SNP0579	76.225	13.473	0.0005
		16	SNP6144	7.443	16.797	0.0001
Suc	April 2021	1	g1A200	73.501	22.039	0.0001
		9	SNP3435	59.043	17.428	******
Suc Loading	April 2021	1	g1A200	73.501	26.781	0.0001
Pi	April 2021	5	SNP1850	67.168	15.074	0.0001
		9	SNP3317	25.462	14.576	0.0005
		16	SNP6185	22.035	13.937	0.0005
TSC	April 2021	12	SNP4559	0.274	14.604	0.0005
		12	SNP4613	12.371	17.837	0.0001
		16	SNP6393	90.602	15.403	0.0001

### 3.7 Identification of genes underlying QTLs

The DNA sequences of the chromosomal region underlying QTLs were retrieved from the genome sequence of clone PB 260. The size of the sequences of a region underlying QTLs ranged from 191 kb to 25,522 kb (Table 5). Ninety-three, sixty-nine, forty-eight, fifty-two and thirty-four putative genes were identified in the chromosomal region underlying QTLs for yield, sucrose, sucrose loading, Pi and TSC, respectively.

**Table 5:** Description of chromosomal region underlying QTLs for yield, sucrose, sucrose loading, Pi and TSC.

Trait	Linkage group	Length of QTL Sequence (kb)	N° of genes
Yield	2	4610	40
	2	826	30
	16	360	23
Sucrose and sucrose loading	1	6397	48
Sucrose	9	286	21
Pi	5	25,522	29
	9	302	8
	16	191	15
TSC	12	356	22
	16	192	12

Among the genes underlying QTLs, only a few that could be linked to the traits under study based on current knowledge of genes encoding regulatory proteins, transcription factors, enzymes, and structural proteins (Supplementary data 11). Concerning latex yield, some genes are involved in fermentation and sugar metabolism, such as *triacylglycerol lipase SDP1-like isoform X2, fructose-1,6-bisphosphatase* and *fructokinase-like 1.* These putative candidate genes may be involved in the metabolic pathways producing the carbon sources for rubber biosynthesis. Another potential candidate gene is *serine/threonine protein phosphatase* which has already been shown to be involved in the mevalonate pathway. Concerning sucrose, alpha-trehalose-phosphate synthase is known to regulate sugar metabolism. In the case of Pi, attention should be paid to genes involved in cellular functions and the six transcription factors. Concerning TSC, *abscisic acid 8′-hydroxylase 4-like* and *ethylene-responsive transcription factor RAP2-10-like* were identified as two putative candidate genes involved in the regulation of ABA level and signalling in response to water stress.

## 4 Discussion

Latex diagnosis is used to monitor the physiological state of rubber trees in order to optimise yield and to minimise the TPD occurrence in estate plantations. For better interpretation of the results, LD should be conducted during the peak production period [18]. This study used latex yield and LD data to form a clonal typology [21]. This classification enabled identification of the production potential of clones with and without stimulation. The use of a high-density map facilitated the detection of QTLs for yield and LD parameters as well as for sucrose loading. QTLs for sucrose loading were analysed for the first time in this study and, to our knowledge, this is the first time that chromosomal regions underlying QTLs for physiological parameters in rubber have been retrieved and the function of several genes associated with these traits.

### 4.1 Clonal typology based on both initial yield and LD can predict the yield capacity of rubber genotypes and their response to ethephon stimulation

The initial yield obtained during the first year of production only provides information on the potential yield of genotypes with high metabolic activity. These genotypes do not require systematic ethephon stimulation to activate their metabolism. This study conducted on a biparental PB 260 x SP 217 population confirmed that Pi content can be used as a simple indicator to identify quick-starter clones in most genotypes. Previous studies already showed the positive correlation between yield and Pi content [15,17,21]. However, these quick-starter clones are prone to tapping panel dryness (TPD) and their yield can decline after a few years of production. On the other hand, some new rubber clones, for example IRR 112 and IRR 118, have both high metabolic activity and low TPD occurrence [13,14]. Interestingly, Junaidi and collaborators showed that low TPD susceptibility is associated with a capacity of regeneration and synthesis of antioxidants, and the same authors suggest that a high concentration of sucrose in latex is the source of energy to support the antioxidant metabolism [19]. In the present study, SP 217 was shown to produce a medium-high rubber yield and medium Pi content, while genotype G097 had a high Pi content of 12.4 mM but produced a low initial yield. This result thus suggests using the clonal typology that combines Pi and sucrose loading calculated from sucrose content and yield would improve the classification of clones based on their latex physiology.

Sucrose is a source of carbon for rubber biosynthesis and of energy for latex regeneration [42]. In this study, sucrose was shown to be high when Pi is low, revealing low consumption of sucrose due to a low latex metabolism. The genotypes with such parameters require activation of their latex metabolism by ethephon stimulation. A group of genotypes with medium Pi and low Suc tended to produce a moderate yield. Suc, Pi and yield are three essential parameters used in the clonal typology [21]. This method led to grouping the genotypes based on their sucrose loading and latex metabolism, and consequently made it possible to identify genotypes that have the capacity to increase their yield under ethephon stimulation.

Clones with low metabolism are often considered to be slow-starter clones, which require ethephon stimulation to increase their metabolism in order to produce latex. In this study, clone SP 217 was shown to be a medium-starter with a very good initial yield. Of the forty-seven genotypes with high sucrose content and low to medium metabolism, ten promising genotypes have a performance that could be improved by ethephon stimulation (Supplementary data 4).

The use of initial latex yield and latex diagnosis-based clonal typology is a way to shorten breeding programmes by predicting the yield capacity and the response to ethephon stimulation of rubber genotypes from an F1 population. Clonal typology also makes it possible to predict the intensity of ethephon stimulation best suited to each genotype. This method has been used by plantation companies for many years to adapt their harvesting system to rubber clones in specific agroclimatic conditions.

### 4.2 QTLs associated with physiological and agronomic parameters of latex production revealed a complex genetic basis

QTLs associated with yield and LD parameters were detected on six linkage groups in the present study, in contrast to previous studies [28,30]. Rattanawong and collaborators detected QTLs associated with yield on thirteen LGs using fourteen yield datasets where the most consistent QTL was found on LG 16. In contrast, An and collaborators identified QTLs associated with yield on only four LGs using four yield datasets. Rattanawong and collaborators used the same SSR markers as those used in the present study, so the names of the linkage groups are similar in the two studies, whereas An and collaborators used different markers. QTLs related to latex yield were detected on both LG 2 and LG 16 by Rattanawong and in the present study, and on LG 8 and LG 13 by Rattanawong teams. The heritability for cumulative yield in the present study was considered high, indicating that 92 % of all phenotypic variation for yield is due to variation in genotypes for that trait. In other words, the effect of genetic factors on cumulative yield is stronger than the effect of environmental factors, it is therefore reasonable to identify QTLs on the similar LGs. Nevertheless, QTLs for yield did vary from time to time, thereby revealing that the environment has an important effect. In another study, QTL analysis using a genetic map generated with SNP markers, successfully identified some significant QTLs for yield, but the position of the LG changed from time to time [30].

Concerning LD parameters, we observed seven QTLs located on five LGs, while Rattanawong's team detected twelve QTLs on eight LGs. No common QTLs for sucrose were were found in the two studies, but both located only a small number of QTLs: two on LG1 and LG9 and one on LG16, respectively. For Pi, three QTLs were detected in the present study and seven in Rattanawong's studies, one QTL being on the same linkage group (LG16), which is the most reliable QTL in Rattanawong's paper. The difference observed between these two studies can have several explanations. Rattanawong and collaborators used several datasets with a small number of genotypes and conducted LD analysis at different times of the year. Indeed, latex diagnosis is affected by the status of canopy as well as by environment factors, which have a dramatic influence on sucrose content and on metabolic activity (Pi) in laticifers. That is why in the present study, particular care was taken to perform LD analysis during the peak production period with a full canopy to avoid any variation in sucrose content due to this environmental factor.

Interestingly, a QTL was found in the same position (on LG 16) for Pi and yield, this suggests that one or several genes underlying this QTL influenced these traits through shared or separate molecular mechanisms. Thanks to the high density of markers, the characterisation of genomic sequences surrounding these QTLs should lead to a better understanding of this mechanism.

### 4.3 The high-density map and the size of the phenotyped population led to more precise detection of QTLs and identification of genes underlying QTLs

Six main studies focussed on detecting QTLs for growth, latex yield and latex physiological traits with high-density map have been published to date (Table 6). The first high-density genetic map of *Hevea brasiliensis* generated an average marker density of 1.90 cM using SNPs from RNA-seq data [51]. More recently, genotyping by sequencing (GBS) and specific locus amplified fragment sequencing (SLAF-seq) were attempted in three studies [30,33,52]. GBS is a rapid and efficient method that can simultaneously detect and score many SNP markers. This technique has been used to construct genetic linkage maps in several plant species [47,[53], [54], [55], [56], [57]]. The genetic distance between the markers is comparable in this study (0.6 cM) and the studies performed by the teams of An (0.59 cM) and Xia (0.3–0.97 cM) and was improved compared to studies conducted by Shearman's (1.9 cM) and Pootakham's (0.89 cM) teams. Moreover, population size is also an important factor that could affect the results of QTL mapping. Precision and the ability to detect QTLs can be improved by increasing the size of population used for the study. Pootakham and collaborators used two populations consisting of respectively, 118 and 79 offspring to construct a high-density integrated linkage map, while a larger population was used by the An et al. (206 genotypes), Xia et al. (187 genotypes) and in the present study (189 genotypes) [30,33,52].

**Table 6:** Summary list of main genetic studies that used high-density map for traits associated with latex yield.

Parent clones	N° of individuals	N° of markers	Genetic distance (cM)	Trait	Reference
RRIM 600 x RRII 105	149	12,326	1.9	Growth, latex yield	[51]
BPM 24 x RRIM 600 and BPM 24 x RRIC 110	118 and 79	2321	0.89	–	[52]
Yunyan277-5 x IAN 873	187	6940	0.3–0.97	Latex yield	[33]
CATAS 8-79 x MT/C/11 9/67	206	4543	0.59	Growth, latex yield	[30]
IAN 873 × REYAN 106	214	203,124		Growth	[32]
PB 260 x SP 217	189	3379	0.6	Latex yield, Suc, Pi, TSC	This study

High-density map makes it possible to map the QTLs and candidate genes. Xia and collaborators identified highly promising candidate genes associated with the dry latex yield [33]. In the present work, the large number of genes underlying QTLs made it difficult to identify candidate genes precisely. The use of the genome sequence of clone PB 260 was expected to improve the detection of candidate genes. Several functions of genes are related to yield, sucrose, and TSC respectively. Despite the very large span of genome regions, one gene for fermentation (*triacylglycerol lipase SDP1-like isoform X2*0) and two genes for sugar metabolism (*fructose-1,6-bisphosphatase* and *fructokinase-like* 1) were identified for yield. In addition to sugar metabolism, fermentation has long been known to be an alternative way to support rubber biosynthesis [58]. Ethanol and latex fermentation is suggested to provide energy for rubber biosynthesis [59]. *HbERF-VIIa12* gene orthologue to *RAP2.12* is highly expressed in latex and could play role in hypoxia response [60]. Putative candidate genes related to water stress for TSC are relevant since TSC depends on the amount of water in the latex. The regulation of ABA concentration by *abscisic acid 8′-hydroxylase* should occur after tapping and the resulting osmotic stress caused by the flow of latex. *Ethylene-responsive transcription factor RAP2-10* belongs to the DREB family, which is a transcription factor involved in signalling in response to water stress.

## 5 Conclusions

To conclude, this paper reports on the successful use of LD in breeding and genetic studies. Yield, Pi, and sucrose data helped categorise genotypes. Conventional breeding has led to the selection of quick-starter clones based on their high yield during the first years of production. This clonal typology is a useful tool for breeders to select genotypes with the capacity to improve their yield through ethephon stimulation. Performing LD in the peak production period should improve the detection of QTLs and several putative candidate genes appear promising with respect to the traits studied here. QTLs for sucrose loading were identified for the first time. This result now needs to be confirmed using other populations in similar experimental conditions. The identification of these candidate genes needs to be improved using other approaches in order to advance our understanding of the molecular mechanisms involved in latex production and to identify specific markers to be used in marker-assisted selection.

## Data availability statement

All raw data used in the present study are available as Supplementary Data.

## CRediT authorship contribution statement

**Sigit Ismawanto:** Writing – review & editing, Writing – original draft, Resources, Methodology, Investigation, Funding acquisition, Formal analysis, Data curation. **Martini Aji:** Writing – review & editing, Investigation, Formal analysis. **David Lopez:** Writing – review & editing, Writing – original draft, Validation, Methodology, Investigation, Formal analysis, Data curation, Conceptualization. **Pierre Mournet:** Writing – review & editing, Writing – original draft, Validation, Resources, Methodology, Investigation, Formal analysis, Data curation, Conceptualization. **Eric Gohet:** Writing – review & editing, Validation, Supervision, Formal analysis, Data curation, Conceptualization. **Afdholiatus Syafaah:** Writing – review & editing, Formal analysis. **Florelle Bonal:** Writing – review & editing, Methodology, Formal analysis, Conceptualization. **Fetrina Oktavia:** Writing – review & editing, Supervision, Resources, Project administration, Funding acquisition. **Taryono:** Writing – review & editing, Supervision, Writing – review & editing, Supervision. **Siti Subandiyah:** Writing – review & editing, Supervision. **Pascal Montoro:** Writing – review & editing, Writing – original draft, Validation, Supervision, Resources, Project administration, Methodology, Funding acquisition, Formal analysis, Data curation, Conceptualization.

## Declaration of competing interest

The authors declare that they have no known competing financial interests or personal relationships that could have appeared to influence the work reported in this paper.
